# TET2 Inhibits Differentiation of Embryonic Stem Cells but Does Not Overcome Methylation-Induced Gene Silencing

**DOI:** 10.1155/2014/986571

**Published:** 2014-08-25

**Authors:** Louis Norman, Paul Tarrant, Timothy Chevassut

**Affiliations:** ^1^Medical Research Building, Brighton and Sussex Medical School, Sussex University, Falmer, Brighton BN1 9PS, UK; ^2^Royal Sussex County Hospital, Brighton and Sussex University Hospitals NHS Trust, Brighton BN2 5BE, UK

## Abstract

TET2 is a methylcytosine dioxygenase that is frequently mutated in myeloid malignancies, notably myelodysplasia and acute myeloid leukemia. TET2 catalyses the conversion of 5′-methylcytosine to 5′-hydroxymethylcytosine within DNA and has been implicated in the process of genomic demethylation. However, the mechanism by which TET2 loss of function results in hematopoietic dysplasia and leukemogenesis is poorly understood. Here, we show that TET2 is expressed in undifferentiated embryonic stem cells and that its knockdown results in reduction of 5′-hydroxymethylcytosine in genomic DNA. We also present DNA methylation data from bone marrow samples obtained from patients with TET2-mutated myelodysplasia. Based on these findings, we sought to identify the role of TET2 in regulating pluripotency and differentiation. We show that overexpression of TET2 in a stably integrated transgene leads to increased alkaline phosphatase expression in differentiating ES cells and impaired differentiation in methylcellulose culture. We speculate that this effect is due to TET2-mediated expression of stem cell genes in ES cells via hydroxylation of 5′-methylcytosines at key promoter sequences within genomic DNA. This leads to relative hypomethylation of gene promoters as 5′-hydroxymethylcytosine is not a substrate for DNMT1-mediated maintenance methylation. We sought to test this hypothesis by cotransfecting the TET2 gene with methylated reporter genes. The results of these experiments are presented.

## 1. Introduction

### 1.1. *TET* Genes, Function, and Background

The family of* TET* (ten-eleven translocations) genes comprise three members,* TET1*,* TET2*, and* TET3,* each with high level of homology to one another.* TET2,* at locus 4q24, has three distinct variant forms from alternative splicing, each of which possesses 2-oxoglutarate- (2OG-) Fe(II) dependent oxygenase activity that is dependent on *α*-ketoglutarate and Fe^2+^ ion as cofactors [1, 2]. TET2 acts functionally to catalyse the base conversion of 5-methylcytosine (5^m^C) DNA to 5-hydroxymethyl-cytosine (5^hm^C) [3–7]. Other 2OG-Fe(II)-dependent enzymes are common in humans with a broad spectrum of functions including DNA repair, hypoxia sensing, histone demethylation, fatty-acid metabolism, and collagen stabilization [8]. In recent studies He et al. and Ito et al. used thin layer chromatography (TLC) and mass spectrometry to show that TET2 has further oxidative activity to convert 5^hm^C to 5-carboxylcytosine (5^ca^C) in HEK293T cells, although the other expected intermediate, 5-formylcytosine (5^f^C), was only observed in mouse embryonic stem (ES) cells in the study by Ito et al. [9] and He et al. [10]. 5^ca^C is a relatively stable base modification and an active demethylation process was seen involving thymine-DNA-glycosylase (TDG) base excision repair [10]. Recent evidence suggests that 5^hm^C can also be dehydroxymethylated to cytosine by the* de novo* methyltransferase proteins DNMT3A and DNMT3B although this needs to be confirmed by other studies [11]. Thus, TET2 plays a central role in the biology of cytosine methylation. However, the functional role of TET2 in the cell and the importance of its product 5^hm^C to transcriptional activity of genes remain to be elucidated.

### 1.2. TET2 in ES Cells and Mutations in MDS/AML

*TET2* mutation is enriched amongst cases of myeloid malignancy occurring in approximately 12% of acute myeloid leukemia (AML) cases and up to 58% of mixed myeloproliferative neoplasms (MPN) and myelodysplastic syndromes (MDS) [12–14]. Therefore, it is likely that TET2 plays an important role in regulation of hematopoietic differentiation and may even serve as a prognostic tool in myeloid malignancies. AML is characterized by enhanced proliferation and impaired differentiation of myeloid progenitors, resulting in clonal populations of immature hematologic blasts. Furthermore specific AML subgroups show patterns of dysregulated DNA methylation patterns, which can be correlated with patient survival [4]. The majority of* TET2* mutations recorded in patients with myeloid malignancy are within regions critical for enzymatic activity suggesting they are loss of function mutations and hence TET2 is seen to be having a tumour suppressor role [1, 3].

The clinical outcome of patients with a mutation in* TET2* remains contentious. In AML patients, one study found a decreased survival rate in* TET2* mutants when compared to a wild-type group [15], whereas another study found no significant impact of mutation status on clinical outcome, but mutation was strongly associated to a mutation in the* NPM1* gene, which is also implicated in AML [16, 17]. In MDS patients the 5-year survival rate was 76.9% for* TET2* mutants against 18.3% for wild-type, indicating a mutation as a marker for good prognosis [18]. Additionally, Smith et al. could find no prognostic significance for* TET2* mutation when patients were clustered into WHO subtypes, scored using the International Prognostic Scoring System, or grouped into cytogenic status or progression to AML [19].

The isocitrate dehydrogenase enzyme* IDH1* and its mitochondrial homologue* IDH2* are responsible for converting isocitrate to *α*-ketoglutarate in the Krebs/TCA cycle. Heterozygous gain of function mutations of* IDH1* and* IDH2* has been shown to convert isocitrate into 2-hydroxyglutarate (2-HG) which competes with *α*-ketoglutarate to inhibit TET activity [4]. Various similarities exist between* TET2* mutant and* IDH1/2* mutant phenotypes including increased 5^m^C in cultured cells and an increase in progenitor cell numbers and stem cells markers [4]. These findings provide important insights into a possible common mechanism of epigenetic disruption that may lead to AML or precursor diseases such as MDS.

### 1.3. Methylcytosine, Hydroxymethylcytosine, and Its Effect on Promoter Activity

The total 5^m^C content of the human genome is 1–3% of all cytosine bases [3, 20–22] occurring almost exclusively at CpG dinucleotides. Clusters of CpG sites occurring at higher than expected frequency within genomic regions are termed CpG islands (CGIs) which are generally hypomethylated within cells. Around 65% of CGIs localise around the point of gene promoter regions including all constitutively expressed genes [23]. Aberrant methylation of CGIs leads to stable transcriptional repression and is thought to be important in silencing tumour suppressor genes in some conditions such as MDS. CGI methylation at promoter regions upstream of genes has the greatest effect on transcription via prevention of elongation of the transcript [20]. Some evidence suggest that CGI methylation within exonic regions of genes is permissive of transcription and does not block initiation or elongation [24]. The recent discovery of cytosine hydroxymethylation raises the possibility that methylation at CGIs can be reversed via active cytosine demethylation involving TET proteins leading to the reactivation of transcriptionally silenced promoters [9, 10]. Furthermore new evidence on induced pluripotent stem cells (iPSCs) suggests that the 5^hm^C base modification not only is an intermediate within cytosine demethylation, but acts as an epigenetic mark in itself, possibly aiding in recruitment of factors that promote chromatin remodeling [25, 26].

We show here that levels of 5^hm^C are reduced by TET2 knockdown in ES cells and in* TET2* mutated MDS bone marrow samples. We investigated the phenotypic effects of* TET2* overexpression on differentiation of murine ES cells using stable transfectants. Finally, using reported genes, we show that transient overexpression of TET2 has no significant effect on the transcriptional activity of methylated promoters.

## 2. Results

### 2.1. Differentiation of ES Cells Leads to Decreased 5^hm^C

**Figure 1:**
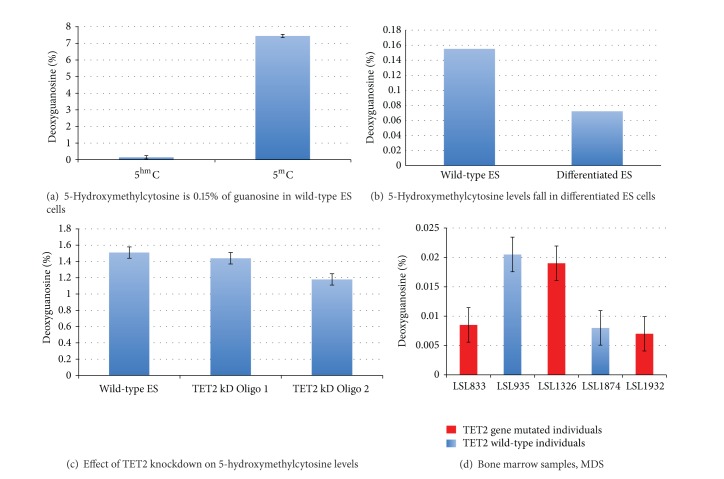
Hydroxymethylation status in ES cells under differentiation and TET2 knockdown and in patient samples. HPLC mass spectrometry was used to determine the levels of methylcytosine (5^m^C) and hydroxymethylcytosine (5^hm^C) expressed as a percentage of total guanosine bases. All experiments were in triplicate and averaged, unless otherwise stated. (a) 5′-hydroxymethylcytosine is 0.15% of wild-type ES cells. Levels of 5^hm^C were compared to levels of 5^m^C in undifferentiated E14 ES cells, grown in the presence of LIF to maintain an undifferentiated state; levels were 0.15% and 7.5%, respectively. (b) 5′-hydroxymethylcytosine levels fall in differentiated ES cells. 5^hm^C levels dropped by approximately 50% from 0.15% to 0.076% relative to guanosine bases upon differentiation via the removal of LIF from the growth media. 5^hm^C levels were determined on day 5 after removal of LIF from the growth medium. (c) Effect of TET2 knockdown on 5′-hydroxymethylcytosine levels. Small inhibitory RNA (siRNA) knockdown of TET2 using a control scrambled oligonucleotide (wild-type ES), and two specific TET2 oligonucleotides (Oligo 1 and Oligo 2). Oligo 1 showed no significant knockdown of 5^hm^C levels compared to the control, whereas Oligo 2 showed reduced levels of 5^hm^C upon HPLC mass spectrometric analysis of around 18% compared to control. (d) Bone marrow samples from patients with myelodysplasia. Marrow samples from myelodysplastic syndrome (MDS) patients were analysed with the mass spectrometer for 5^hm^C status. Patients were genotyped for mutations in the* TET2* gene locus and any mutants were found to be heterozygous for the* TET2* gene.

We sought to investigate the levels of 5^m^C and 5^hm^C in undifferentiated murine ES cells using HPLC mass spectrometry. We found levels of 7.5% for 5^m^C and 0.15% for 5^hm^C relative to guanosine bases demonstrating that 5^m^C is present in ES cells at approximately 50-fold greater levels than 5^hm^C (Figure 1(a)).

Next we sought to investigate what happens to levels of 5^hm^C during induction of differentiation. We compared levels of 5^hm^C in undifferentiated ES cells grown in the presence of leukemia inhibitory factor (LIF) and in differentiated cells 5 days post-LIF withdrawal (Figure 1(b)). We found that levels of 5^hm^C fall by approximately 50% from 0.155% to 0.072% relative to guanosine bases upon differentiation. This result suggests that LIF withdrawal induces downregulation of one or more of the TET family genes leading to a reduction in conversion of 5^m^C to 5^hm^C.

### 2.2. Knockdown of TET2 in ES Cells Causes Reduced Levels of 5^hm^C

Based on this finding we performed siRNA knockdown experiments of the TET2 gene. Using two different gene-specific siRNA oligos in triplicate knockdown experiments we measured reductions in 5^hm^C levels of 5% and 20%, respectively, compared with a scrambled control (Figure 1(c)). These results provide evidence that TET2, like TET1, catalyses the conversion of 5^m^C to 5^hm^C.

### 2.3. Measurement of 5^hm^C Levels in* TET2* Mutated MDS/AML Patient Marrow Samples

Genomic DNA analysis using mass spectrometry of five patients with myeloid malignancy failed to identify a clear association between the* TET2* mutational status and 5^hm^C levels (Figure 1(d)). Patient samples were genotyped to identify mutations within the* TET2* gene locus; any patients found to have a mutation were heterozygous wild-type. While this result is based on a small number of samples and was not correlated with the size of the malignant clone or the nature of the genetic mutation, it nonetheless shows that global deficiency of 5^hm^C is not sufficient on its own to cause disease. However, it does not preclude the possibility that localised loss of 5^hm^C at specific gene sites has occurred in* TET2* mutated disease.

### 2.4. Stable Transfection of Murine ES Cells with a* TET2*-Containing Plasmid Vector Leads to Slight Increased 5^hm^C Level Immunostaining

**Figure 2:**
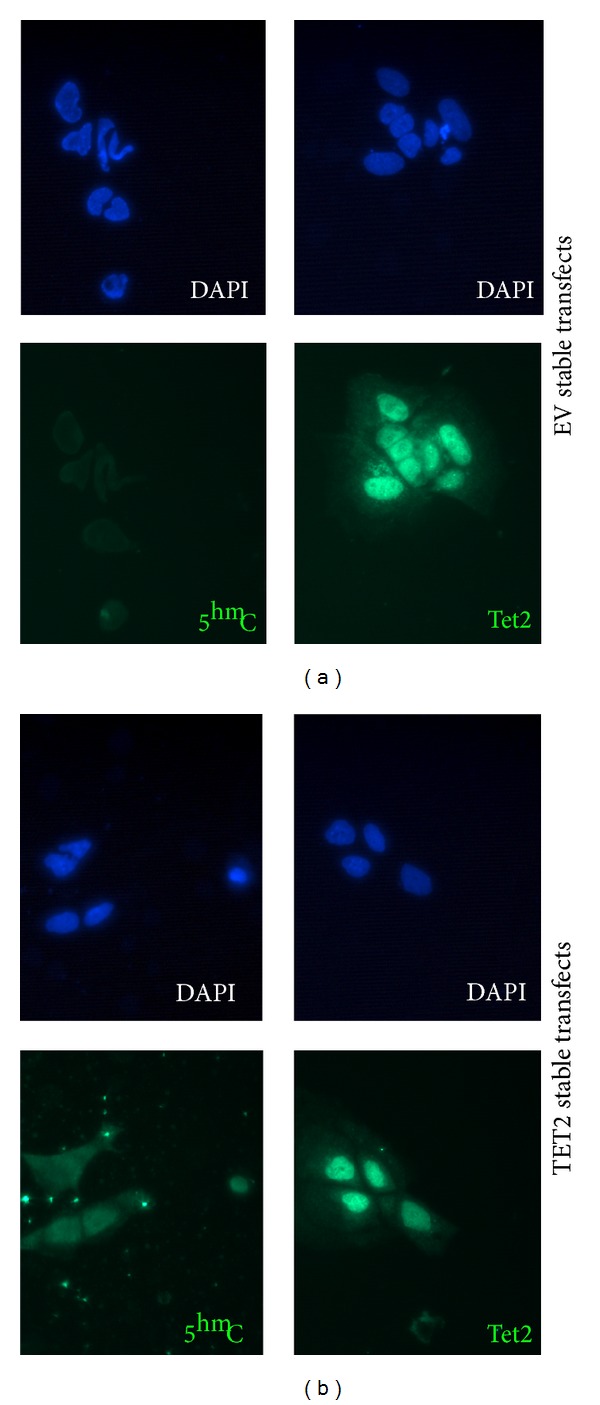
Stable transfection of murine ES cells with a pCDNA-TET2 plasmid vector leads to slight increased immunostaining for 5^hm^C. Stably transfected E14 murine ES cells were grown to confluency and analysed by immunocytochemistry for 5^hm^C, TET2, and 4′,6-diamidino-2-phenylindole (DAPI) as a nuclear-identifying control. Empty vector (EV) stable transfect cells are shown in (a) and TET2 stable transfect cells are shown in (b).

E14 murine ES cells were stably transfected with TET2-pCDNA3 plasmid vector (TET2 stable transfects) or empty pCDNA3 plasmid as a control (EV stable transfects) in triplicate. Selection in G418 antibiotic confirmed the presence of the incorporated plasmid DNA and surviving colonies were grown to confluency.  Figure 2 shows that TET2 was present to a relatively similar degree in both EV and TET2 stable transfects and was strongly associated to the nuclear region of cells. 5^hm^C levels were slightly raised in TET2 stable transfects.

### 2.5. Stable Transfection of TET2-pCDNA3 Vector Reduces ES Cellular Differentiation as Observed by Leukocyte Alkaline Phosphatase Analysis

**Figure 3:**
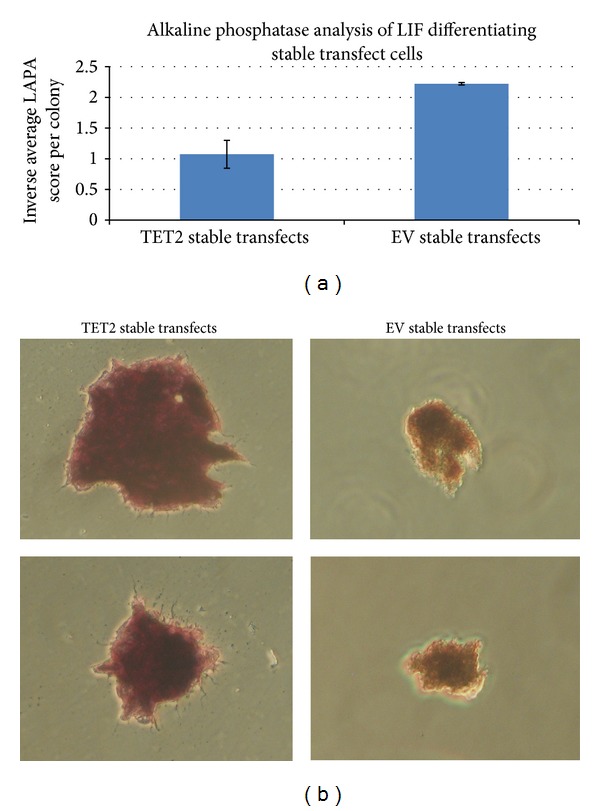
Alkaline phosphatase analysis of stably transfected ES cells at 7 days post-LIF removal. Once at confluency stably transfected pCDNA3 and TET2-pCDNA3 E14 ES cells were plated at 1000 cells per plate on gelatin coated 10 cm tissue culture plates in triplicate. 72 hours after plating LIF was removed from the media and colonies were left to grow for a further 7 days. Analysis of alkaline phosphatase activity was conducted using the Alkaline Phosphatase Leukocyte kit (Sigma-Aldrich) and each colony was given a leukocyte alkaline phosphatase (LAPA) score. (a) Average LAPA scores of stably transfected ES cells at 7 days post-LIF removal. A total of 159 colonies were analysed for the TET2-pCDNA3 condition and 128 for the pCDNA3 condition. The LAPA score for TET2-pCDNA3 was 0.9 (inverse was 1.07), whereas pCDNA3 stable transfects had an average LAPA score of 0.45 (inverse was 2.22), showing that the TET2-pCDNA3 colonies were 50% less differentiated than the control colonies. (b) shows colonies at 7 days post-LIF removal that have been stained for alkaline phosphatase (AP) activity.

A differentiation assay was conducted by removing LIF from the media and relative differentiation measured using alkaline phosphatase (AP) analysis, where each colony is attributed to a leukocyte AP analysis (LAPA) score. Reducing alkaline phosphatase activity is an indicator of increasing differentiation. LAPA scoring of 7 day differentiating cells revealed colonies from the TET2-pCDNA3 condition having an average LAPA score of 0.9 compared to 0.45 for the control group, showing that the TET2-pCDNA3 transfect colonies were on average 50% less differentiated than the control transfect colonies at the time of analysis (Figure 3(a)). A total of 262 colonies were analysed for AP activity across the two conditions.  Figure 3(b) shows typical colonies observed at 7 days of differentiation post-LIF withdrawal and stained for AP activity.

### 2.6. Stable Transfection of Murine ES Cells with a* TET2*-Containing Plasmid Vector Leads to a Slightly Reduced Ability to Differentiate under Methylcellulose + G-CSF

**Figure 4:**
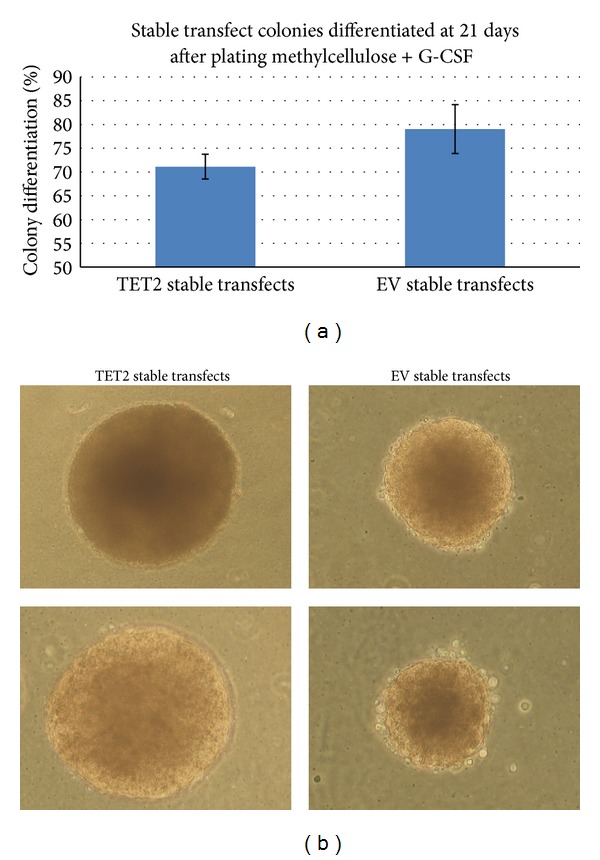
Methylcellulose differentiation of stably transfected ES cells in the presence of hematopoietic cytokines. E14 ES cells were stably transfected as described and grown to confluency. Triplicate 10 cm gelatin coated tissue culture plates containing 4000 cells each were plated of both the TET2-pCDNA3 (TET2 stable transfect) and pCDNA3 control (EV stable transfect). Cells were differentiated using MethoCult GF H4034 (Stem Cell Technologies), a combination of methylcellulose and granulocyte-colony stimulating factor (G-CSF) in the media and colonies analysed according to the manufacturer's instructions at day 22 after plating for differentiation. A total of 229 colonies were classified in the TET2 stable transfect condition and 173 colonies for the EV stable transfect condition. (a) Relative differentiation levels of TET2 stable transfects and EV stable transfects at 22 days under methylcellulose condition. TET2 stable transfects were on average 8% less differentiated at the time of analysis. (b) Photomicrographs of embryoid bodies derived from stably transfected ES cells.

Following this we conducted a further differentiation experiment using methylcellulose with granulocyte-colony stimulating factor (G-CSF). At 22 days under MethoCult + G-CSF differentiation cells showed a slight difference in extent of differentiation, with an indication of a reduction in the percentage of cell colony differentiation seen with the TET2-pCDNA3 vector transfects compared to controls (Figure 4(a)). A total of 412 colonies were scored based on the MethoCult GF H4034 protocol and observed under a light microscope across triplicate experiments of both TET2-pCDNA3 vector and pCDNA3 control stable transfects, respectively. Typical colonies observed are illustrated in Figure 4(b).

### 2.7. Murine ES Cell Differentiation Fails to Overcome Methylation Induced Silencing

**Figure 5:**
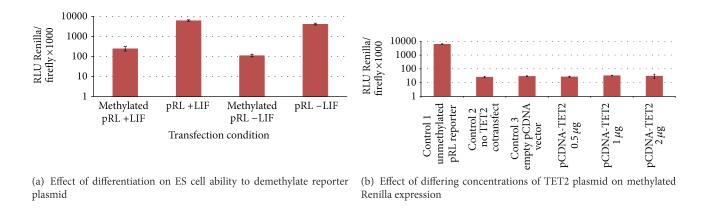
(a) Expression of methylated reporter plasmids is identical in differentiated and undifferentiated ES cells. A Dual Luciferase Reporter Assay (Promega) was modified to determine if E14 murine ES cells undergoing differentiation were more able to demethylate (unsilenced) a reporter gene plasmid. Methylation of the pRL reporter by SssI methylase (Promega M0226S) showed optimal gene silencing, and relative expression of the Renilla Luciferase was compared to the unmodified pGL3 plasmid expression of Firefly Luciferase via a luminometer. E14 cells were plated at 1 × 10^5^ cells per well in a 24-well plate in triplicate experiments and induced to differentiation on day 1 posttransfection by removal of leukemia inhibitory factor (LIF), and transient transfection of both the pGL3 and methylated pRL was conducted using the Lipofectamine LTX (Invitrogen) reagent as per the manufacturer's instructions on day 5 after plating. Analysis of Renilla and Firefly expression was conducted on day 6. Transient transfection of unmodified pRL and pGL3 was used as a control. (a) shows that although methylation clearly had a significant silencing effect on pRL, there was no significant difference seen between the −LIF (differentiating) or +LIF (undifferentiated) conditions on relative expression of pRL. (b) TET2 is unable to overcome silenced expression of a cotransfected methylated Renilla plasmid. Human embryonic kidney (HEK) 293T cells were plated at 3 × 10^4^ cells per well in a 24-well plate in triplicate and transiently transfected using the Lipofectamine LTX (Invitrogen) reagent as per the manufacturer's instruction on day 1 after plating with both Dual Luciferase reporter plasmids and a TET2-pCDNA3 construct plasmid. Control experiments were run in parallel with either no pRL methylation, no pCDNA-TET2 cotransfect, or a pCDNA3 (empty vector) cotransfect. Dual Luciferase experiments were modified by methylation of the pRL reporter by SssI methylase (Promega M0226S), which showed optimal gene silencing, and relative expression of the Renilla Luciferase was compared to the unmodified pGL3 plasmid expression of Firefly Luciferase via a luminometer. Experiments were conducted in triplicate and averaged. Cotransfection of a* TET2*-pCDNA3 plasmid vector at varying concentrations with the Dual Luciferase plasmids does not lead to changes in the unsilencing (demethylation) of the methylated reporter. Control 1 shows again that unmethylated pRL plasmid has much higher expression levels than the methylated form. There was no dose response, even when 2 *μ*g of TET2-pCDNA3 plasmid DNA was added to each well, and there was no difference in Renilla expression between controls 2 and 3 and between those cotransfections that contained the TET2-pCDNA3 plasmid.

pRL (Renilla) and pGL3 (Firefly) Luciferase reporter plasmids were then used to determine the ability of E14 cells to demethylate a previously methylated reporter plasmid. Transient transfections were carried out using methylated or unmethylated pRL reporter plasmid in differentiating (−LIF) or undifferentiating (+LIF) conditions and relative luminescence was compared to the secondary pGL3 reporter that was not methylated (Figure 5(a)). No significant difference was seen between the −LIF and +LIF conditions; however, the methylated pRL condition leads to a significant reduction (of around 96%) in relative Renilla luminescence compared to Firefly. This shows the methylation had successfully silenced the reporter, by reducing the cells ability to express the Renilla protein, but upon differentiation there was no difference in the cells ability to demethylate and hence reexpress the reporter gene.

### 2.8. TET2 Fails to Overcome Silencing of a Methylated Luciferase Gene When Cotransfected into HEK Cells

In order to determine if the concentration of TET2 protein within the cell impacts DNA methylation, transient transfection experiments were performed on HEK293T cells using the pRL reporter plasmid (either methylated or unmethylated) plus differing concentrations of the TET2-pCDNA3 plasmid construct (Figure 5(b)). The unmethylated pRL plasmid control showed high luminescence in comparison with methylated pRL with relative expression reduced by >98%. However, increasing amounts of TET2-pCDNA3 plasmid cotransfected with the pRL reporter failed to demonstrate any significant difference in Renilla expression levels.

## 3. Discussion

Our findings reveal that ES cell differentiation induced by LIF-removal is associated with a 50% reduction in levels of hydroxymethylcytosine that is likely to be due to downregulation of* TET* genes. We also demonstrate that knockdown of TET2 in ES cells leads to reduced levels of 5^hm^C even in the presence of LIF consistent with the finding of others suggesting that TET2 normally converts 5^m^C to 5^hm^C [3–7]. Our observations suggest that* TET2* gene expression is important for maintenance of ES cell pluripotency although the mechanism whereby this occurs is not clear. We speculate that TET proteins prevent silencing of stem cell genes by maintaining promoters in a hypomethylated state. This might also explain why loss of function of TET2 contributes to myeloid malignancies such as MDS and AML due to silencing of tumour suppressor genes via aberrant promoter methylation. In support of this possibility, mutations of* IDH1* and* IDH2* also occur in myeloid malignancies where neomorphic activity of the enzyme results in aberrant generation of 2-hydroxyglutarate, which inhibits the 2-oxoglutarate dependent conversion of 5^m^C to 5^hm^C by TET2 [4, 7].

We studied the association between the* TET2* mutational status and 5^hm^C levels in bone marrow samples obtained from patients with myelodysplasia. We found no clear association between 5^hm^C levels and* TET2* mutational status although importantly the* IDH1/2* mutational status of these samples was not known. Whilst our analysis of patient samples here was limited due to small numbers, we note with interest that other investigators have indeed found that* TET2* and* IDH1/2* mutated bone marrow samples typically have reduced levels of 5^hm^C compared with nonmutated samples from patients with MDS or AML [4].

We sought next to test the hypothesis that* TET2* expression promotes ES cell pluripotency. We find that ES cells carrying a stably integrated* TET2* transgene expressed more alkaline phosphatase following LIF removal than control ES cells carrying the empty vector suggesting that aberrant expression of* TET2* may impair normal ES cell differentiation. Consistent with this result, we find that ES cells carrying the* TET2* transgene yielded more undifferentiated colonies in methylcellulose culture than did ES cells carrying the empty vector suggesting again that* TET2* expression promotes a pluripotent ES cell state.

Based on our findings we hypothesised that TET2 functions by maintaining gene promoters in a hypomethylated state and that this is important for ES cell pluripotency. Our results failed to demonstrate any significant effect of* TET2* cotransfection on luciferase expression suggesting that the TET2 enzyme is unable to reactivate gene silencing due to promoter methylation. However, we were unable to confirm expression of the* TET2* transgene over and above the endogenous gene. Moreover, TET2-mediated “demethylation” may occur via passive loss of methylcytosines during cell division rather than active demethylation of methylated promoters [23]. Transfection experiments in undifferentiated and differentiated ES cells once again failed to demonstrate differential expression of the luciferase reporter genes according to the methylated state of their promoters. Hence, we conclude that TET2 does not directly affect transcriptional activity of genes at methylated promoters. However, it remains distinctly possible that TET2 functions to maintain unmethylated genes in an active state by maintenance of promoter hypomethylation via hydroxylation of methylcytosines leading to passive loss. If so, then we speculate that this action may be important for the role of TET2 in ES cell pluripotency and myeloid tumour suppression.

## 4. Materials and Methods

### 4.1. Cell Culture

Human embryonic kidney (HEK) 293T cells were cultured in Dulbecco's modified eagle medium (DMEM) supplemented with 10% fetal calf serum, 100 *μ*g/mL penicillin, 100 *μ*g/mL streptomycin, and 100 mM L-glutamine. Murine E14 ES cells were cultured in Glasgow Minimum Essential Medium (GMEM) supplemented with 10% fetal calf serum, 100 *μ*g/mL penicillin, 100 *μ*g/mL streptomycin, 100 mM L-glutamine, 100 *μ*g/mL nonessential amino acids (NEAAs), 10 *μ*g/mL 2-mercaptoethanol (2-ME), and 10 *μ*g/mL leukemia inhibitory factor (LIF) (Sigma L5158). Both cell types were cultured in T25 or T75 tissue culture flasks in tissue incubators with 5% CO_2_ at 37°C.

### 4.2. siRNA

siRNA knockdown experiments of TET genes were conducted using Dharmacon siGENOME siRNA duplexes (Thermo Fisher Scientific Inc.) according to the manufacturer's instructions. siRNA molecules against murine TET1 (Cxxc6_1) and TET2 (E130014J05Rik_5 and E130014J05Rik_6) were obtained and the Dharmacon siGENOME nontargeting siRNA number 2 (D-001210-02) was used as a negative control. Knockdown experiments were performed on E14 ES cells cultured in LIF-containing medium on gelatin coated 12-well plates at a density of 1 × 10^5^ cells per well. Serial siRNA transfections were performed using 50 nM siRNA and Lipofectamine RNAiMAX reagent (Invitrogen). Retransfections were performed on preadherent cells at day 2 at a split of 1 : 4 and finally at day 4 at a split of 1 : 2 in 6-well plates. Cells were harvested at day 5 for HPLC mass spectrometry analyses.

### 4.3. HPLC Mass Spectrometry Analysis

To measure levels of 5^hm^C and 5^m^C a high-performance liquid chromatography (HPLC) mass spectrometry technique was used, measuring levels of 5-hydroxymethyl-deoxycytidine (5^hmd^C) and 5-methyl-deoxycytidine (5^md^C), respectively. DNA was extracted and digested using benzonase and alkaline phosphatase and a 5 *μ*L sample used for analysis. The HPLC configuration was as follows: Phenomenex Gemini C18 column (150 × 2.0 mm ID) with 3 *μ*m size particle guard cartridge, mobile phase A, 0.1% formic acid B, acetonitrile/0.1% formic acid:

 0–2 mins 1% B,
 2–7 mins 1% to 40% B,
 7–14 m mins 1% B.

The Applied Biosystems QTrap MS/MS detector configuration was as follows: orthogonal electrospray ion source, with parent ion/product ion in the following ratios:

 dG–268.1/152.1,
 5mdC–242.1/126.2,
 5ohmdC–258.2/142.2.

Source temperature was 450°C and a spray voltage of 3 kV was used. Collision gas pressure was 1.5 mTorr. Data was then graphed as a percentage of deoxyguanosine bases.

### 4.4. Immunocytochemistry

Cells were passaged into wells of a 24-well plate at 3000 cells per well containing poly-L-lysine (PLL) coverslips 24 hours prior to immunolabeling. Cells were indirectly labelled using the standard procedure by removing coverslips and washing in phosphate buffered saline (PBS), fixing cells in 4% paraformaldehyde for 20 minutes, rewashing in PBS, and permeabilising cells in 0.4% Triton for 5 minutes before washing again in PBS. The following antibodies were used as per the manufacturer's recommended dilutions and instructions: TET2 protein was labelled with TET2 rabbit IgG (S-13; Santa Cruz Biotechnology Inc.) with Alexa488 goat anti-rabbit (Invitrogen) secondary antibody. 5^hm^C was labelled with 5^hm^C rabbit (Active Motif) with Alexa488 goat anti-rabbit secondary antibody. Coverslips were rinsed again in PBS and then costained with 4′,6-diamidino-2-phenylindole (DAPI) for 5 minutes and then rinsed again. DAPI acts as a nuclear identifying control. Coverslips were placed on Citifluor AF1 mounting medium on glass slides and sealed with nail varnish. Cells were visualized under a DM5000B (Leica) fluorescence microscope and images captured using an attached DPC300FX (Leica) digital camera.

### 4.5. Dual Luciferase Reporter Assay

Dual Luciferase Reporter Assay (Promega) was optimized and conducted as per the manufacturer's instructions, using 20 *μ*L of each reagent for each individual reading at 24 hrs posttransfection. Relative luminescence was measured with a GloMax 20/20 Luminometer on the Dual Glo setting and the ratio of Renilla protein (from the pRL plasmid) to Firefly protein (from the pGL3 plasmid) luminescence was graphed.

Methylation of the Renilla luciferase reporter plasmid was conducted using the SssI methylase (Promega M0226S) as this provided optimal methylation when compared to other methylases when testing comparative luminescence and also with a BstUI restriction digest, which cuts DNA at sites of unmethylated 5′…CGCG…3′ (data not shown).

### 4.6. Stable Transfection and Differentiation

pCDNA3 and TET2-pCDNA3 plasmid constructs were kindly donated by Professor Olivier Barnard of INSERM, Institut Gustave Roussy, Villejuif, France. E14 cells were cultured in gelatin coated plates and transfected using the LipofectamineLTX and PLUS reagents (Invitrogen) with the TET2-pCDNA3 plasmid, pCDNA3 control plasmid or mock transfected, each in triplicate. Incorporation of plasmid DNA was selected using 300 *μ*g mL^−^ Geneticin G418 selection antibiotic (Invitrogen) at 5 days posttransfection for 14 days, from which point 100 *μ*g mL^−^ G418 was used. After the 14-day selection period no mock transfected colonies were apparent.

Myeloid differentiation was measured by colony forming cell assay (CFC), conducted using MethoCult Growth Factor H4034 (Stem Cell Technologies). 3 × 3 cm plates of both TET2-pCDNA3 and pCDNA3 stable transfect cells were grown with GF H4034, with control plates of the stably transfected lines grown without GF H4034. At 22 days, colonies were classified according to the MethoCult GF H4034 protocol and photographed using a light microscope-mounted Canon Powershot G5 with B-52 lens adapter and Carl Zeiss 426126 digital camera adapter.

Alkaline phosphatase analysis of stably transfected differentiating E14 cells by removal of LIF was conducted using Alkaline Phosphatase Leukocyte kit (Sigma-Aldrich). Stably transfected cells were plated onto gelatin coated 10 cm tissue culture plates at 1000 cells per plate in triplicate. Media were changed at 72 hours after plating that did not contain LIF. Cells were stained 7 days after removal of LIF and each colony given a leukocyte alkaline phosphatase activity (LAPA) score according to the protocol. Reference LAPA score colonies were photographed as above.

## References

[1] Mohr F, Döhner K, Buske C, Rawat VP (2011). TET genes: new players in DNA demethylation and important determinants for stemness. *Experimental Hematology*.

[2] Strausberg RL, Feingold EA, Grouse LH (2002). Generation and initial analysis of more than 15,000 full-length human and mouse cDNA sequences. *Proceedings of the National Academy of Sciences of the United States of America*.

[3] Tahiliani M, Koh KP, Shen Y (2009). Conversion of 5-methylcytosine to 5-hydroxymethylcytosine in mammalian DNA by MLL partner TET1. *Science*.

[4] Figueroa ME, Abdel-Wahab O, Lu C (2010). Leukemic IDH1 and IDH2 mutations result in a hypermethylation phenotype, disrupt TET2 function, and impair hematopoietic differentiation. *Cancer Cell*.

[5] Ito S, Dalessio AC, Taranova OV, Hong K, Sowers LC, Zhang Y (2010). Role of tet proteins in 5mC to 5hmC conversion, ES-cell self-renewal and inner cell mass specification. *Nature*.

[6] Ko M, Huang Y, Jankowska AM (2010). Impaired hydroxylation of 5-methylcytosine in myeloid cancers with mutant TET2. *Nature*.

[7] Xu W, Yang H, Liu Y (2011). Oncometabolite 2-hydroxyglutarate is a competitive inhibitor of α-ketoglutarate-dependent dioxygenases. *Cancer Cell*.

[8] Loenarz C, Schofield CJ (2009). Oxygenase catalyzed 5-methylcytosine hydroxylation. *Chemistry and Biology*.

[9] Ito S, Shen L, Dai Q (2011). Tet proteins can convert 5-methylcytosine to 5-formylcytosine and 5-carboxylcytosine. *Science*.

[10] He Y, Li B, Li Z (2011). Tet-mediated formation of 5-carboxylcytosine and its excision by TDG in mammalian DNA. *Science*.

[11] Chen C, Wang K, Shen C-K (2012). The mammalian *de novo* DNA methyltransferases DNMT3A and DNMT3B are also DNA 5-hydroxymethylcytosine dehydroxymethylases. *Journal of Biological Chemistry*.

[12] Langemeijer SMC, Kuiper RP, Berends M (2009). Acquired mutations in TET2 are common in myelodysplastic syndromes. *Nature Genetics*.

[13] Hellström-Lindberg E (2010). Significance of JAK2 and TET2 mutations in myelodysplastic syndromes. *Blood Reviews*.

[14] Jankowska AM, Szpurka H, Tiu RV (2009). Loss of heterozygosity 4q24 and TET2 mutations associated with myelodysplastic/myeloproliferative neoplasms. *Blood*.

[15] Abdel-Wahab O, Mullally A, Hedvat C (2009). Genetic characterization of TET1, TET2, and TET3 alterations in myeloid malignancies. *Blood*.

[16] Nibourel O, Kosmider O, Cheok M (2010). Incidence and prognostic value of TET2 alterations in de novo acute myeloid leukemia achieving complete remission. *Blood*.

[17] Garzon R, Garofalo M, Martelli MP (2008). Distinctive microRNA signature of acute myeloid leukemia bearing cytoplasmic mutated nucleophosmin. *Proceedings of the National Academy of Sciences of the United States of America*.

[18] Kosmider O, Gelsi-Boyer V, Cheok M (2009). TET2 mutation is an independent favorable prognostic factor in myelodysplastic syndromes (MDSs). *Blood*.

[19] Smith AE, Mohamedali AM, Kulasekararaj A (2010). Next-generation sequencing of the *TET2* gene in 355 MDS and CMMLpatients reveals low-abundance mutant clones with early origins, but indicates no definite prognostic value. *Blood*.

[20] Patra SK, Patra A, Rizzi F, Ghosh TC, Bettuzzi S (2008). Demethylation of (Cytosine-5-C-methyl) DNA and regulation of transcription in the epigenetic pathways of cancer development. *Cancer and Metastasis Reviews*.

[21] Suzuki MM, Bird A (2008). DNA methylation landscapes: provocative insights from epigenomics. *Nature Reviews Genetics*.

[22] Lister R, Pelizzola M, Dowen RH (2009). Human DNA methylomes at base resolution show widespread epigenomic differences. *Nature*.

[23] Illingworth RS, Bird AP (2009). CpG islands—“a rough guide”. *FEBS Letters*.

[24] Bender CM, Gonzalgo ML, Gonzales FA, Nguyen CT, Robertson KD, Jones PA (1999). Roles of cell division and gene transcription in the methylation of CpG islands. *Molecular and Cellular Biology*.

[25] Doege CA, Inoue K, Yamashita T (2012). Early-stage epigenetic modification during somatic cell reprogramming by Parp1 and Tet2. *Nature*.

[26] Loh KM, Lim B (2012). Epigenetics: actors in the cell reprogramming drama. *Nature*.

